# Significance of ossificated ungular cartilages regarding the performance of cold-blooded trotters

**DOI:** 10.1186/s13028-014-0074-y

**Published:** 2014-10-25

**Authors:** Ulf O Hedenström, Ove S Wattle

**Affiliations:** National Equine Education Centre, Wången AB, SE-83593 Alsen, Sweden; Division of Diagnostics and Large Animal, Department of Clinical Sciences, Swedish University of Agricultural Sciences, SE-75007 Uppsala, Sweden

**Keywords:** Equine, Horse, Ossification, Performance, Ungular cartilage, Collateral cartilages, Side bones, Sidebones, Radiology

## Abstract

**Background:**

Ossification of the ungular cartilages (OUC) in the foot of horses has been studied for more than 100 years. There is a high heritability of this condition but its clinical relevance has remained questionable. Nevertheless, modern equine orthopedic literature ranks OUC as one of top 10 causes of lameness in cold-blooded trotters and stallions of these breeds are excluded from breeding if they have more than mild levels of side bones. Cold-blooded trotters have been used for racing for many decades and official sports data have been available since 1923. A decreased performance is often the only obvious clinical sign noticed by trainers and owners motivating them to seek professional help from veterinarians and farriers. By comparing various performance parameters in Swedish-Norwegian cold-blooded trotters without and with different grades of OUC, we aimed to determine the clinical relevance of ossified hoof cartilages in a population of high-performance horses.

Front hooves from 649 Swedish-Norwegian cold-blooded trotters were evaluated radiologically regarding OUC. Breeding index and official sports data originating from strict protocols kept by groups of officials in trotting associations was used for comparison of performance of these horses that together had competed in more than 23,000 races between 1973 and 2009. Generalized linear mixed models were used for the statistical analyses. The response variable was modeled using ordinal logistic models with a multinomial distribution and a cumulative logit link function. The horse was used as a random factor.

**Results:**

Significant effects of gender on performance were demonstrated, but no correlations were found between different positions nor grades of ossified ungular cartilage and number of starts, running pace, race winnings, number of races completed in a regular gait.

**Conclusions:**

Ossification of the ungular cartilages does not cause decreased performance in cold-blooded trotters and is therefore most likely not a cause of clinical or subclinical lameness in this breed. Results from this study can assist equine professionals in evaluating and interpreting the clinical relevance of radiological findings on ossified hoof cartilage among heavy and high-performing horses.

## Background

Ossification of the ungular cartilages (OUC) in the foot of horses has been studied in different breeds for more than 100 years and the high heritability of this condition is well known [1-5]. For many decades, a number of breed clubs in Sweden and Norway have excluded stallions with high grades of OUC from their breeding programs. However, this measure is frequently debated among Swedish and Norwegian breeders, horse owners, farriers and veterinarians. One breeding organization, Swedish Ardennes, has recently ceased evaluating OUC when classifying stallions before breeding, since Tullberg and Wattle [6] showed that at least 80% of the horses had OUC despite 70 years of breeding to lower its incidence in the population. Furthermore, it has not yet been shown that OUC, unless fractured, is a cause of lameness in the Swedish Ardennes horses. For other breeds too, there are only vague indications that OUC, if not fractured [7], can cause lameness. In a study of 21 Finnhorses, Ruohoniemi *et al.* [8] suggested that the performance of four of the horses might have been affected by OUC. Dyson and Nagy [9] reported significant association between extensively ossified cartilages of the foot and collateral ligament and distal phalanx injury. The authors proposed that a possible clinically significant ossification was found in 13% of 462 cases [9]. Mair and Sherlock [10] described an association between injuries to multiple structures of the foot in 15 horses with high grades of OUC and another study on cadaver feet from six Finnhorses by Ruohoniemi *et al.* [11] proposed incomplete fusion lines as a potential relevant clinical finding and highlighted separate centers of ossification and medial OUC as possibly more clinically significant. Neither Dyson and Nagy [9], Mair and Sherlock [10] nor Ruohoniemi *et al.* [11] showed a correlation between clinical signs and OUC alone.

The Swedish-Norwegian cold-blooded trotter and the Finnish cold-blooded trotter are similar breeds, occasionally racing together, both slightly heavier than the standard- bred trotter. Among professional trainers, horses’ locomotion problems at high speed is an important clinical finding anecdotally suggested to be caused by OUC, but there are no studies including high speed work or other performance data available. Nevertheless, modern equine orthopaedic literature ranks OUC as one of top 10 causes of lameness in cold-blooded trotters [12]. Based on governmental animal welfare regulations [13] Swedish, Norwegian and Finnish trotting associations consider OUC a pathological condition and the annual Scandinavian breeding evaluations exclude 1–2 high-performance stallions every five years due to high grades of OUC. However, the breeding plans are continuously updated on the basis of new data.

Using a 0–5 point scale, Ruohoniemi *et al.* [5] showed that the incidence of any grade of OUC was 80% in Finnhorses while the incidence of OUC ≥ grade 3–5 was 35%. In a study of 2-year-old Swedish-Norwegian cold-blooded trotters, 70% had some grade of OUC while 13% had OUC ≥ grade 3 [4].

The cold-blooded trotter has a high performance potential over a long career, from 2 to 15 years of age, and the capacity to be frequently trained and raced at high speed (>10 m/s) on different, but often hard, surfaces and in differently cambered curves. Many of these trotters are trained frequently from 1.5 years of age. A slightly decreased performance in training and racing is often the only or most obvious sign for which trainers and owners seek professional help. The availability of reliable sports data [14] and many high performing individuals makes the cold-blooded trotter a good homogeneous population for clinical retrospective research. Many of these horses follow racing with an alternative career and are often kept as pleasure horses for numerous years. This makes it possible to locate and re-examine a large number of horses years after they have finished their racing career.

The aim of this study was to compare various performance parameters in Swedish-Norwegian cold-blooded trotters without and with different grades of OUC.

Our hypothesis was that OUC somehow affects the performance of cold-blooded trotters.

## Methods

This project was approved by the Uppsala Animal Ethics Committee, Dnr C 88/6.

Breeding index or Best Linear Unbiased Prediction (BLUP) [15,16] was used for comparison of performance potential with the total population of cold-blooded trotters and between horses examined with different grades of OUC. A BLUP value of 100 is an objective mean value regarding breeding and selected performance markers in a continuously updated five year period of all horses in a breed. Individual data, including BLUP, were collected from official Swedish and Norwegian sports databases during 2009 [14], when more than 99% of included horses had completed their racing careers.

Front hooves from 649 Swedish-Norwegian cold-blooded trotters, born between 1968–1999, were evaluated for OUC. One examined horse was excluded because of uncertain identification. For 197 mares and 211 males (geldings and stallions), all born in 1995, the radiographs were taken at age 2.5 ± 0.25 years, in connection with the study reported by Holm *et al.* [4]. Twelve stallions were radiographed in connection with Norwegian pre-breeding evaluations (mean age 5, median 4 years), while 96 mares and 133 geldings/stallions (mean age 5, median 6 years) were radiographed in connection with gatherings of cold-blooded trotters through open invitations to horse owners living in a 160,000 km^2^ area in the central third of Sweden. All horses in this study were over 1-year old and 95% were 2 years or older at the time of OUC evaluation. Horses <2-years-old were included since training on hard surfaces normally starts as yearlings in this breed which may affect both development of distal phalanx, OUC and pathological conditions of the front feet. In addition, 147 of the horses were re-examined, mean 9 and median 8 years after the first occasion and are also included in a study of ossification development over time [17]. For these horses, the grade of OUC at the last examination was the OUC grade used in this study.

**Figure 1:**
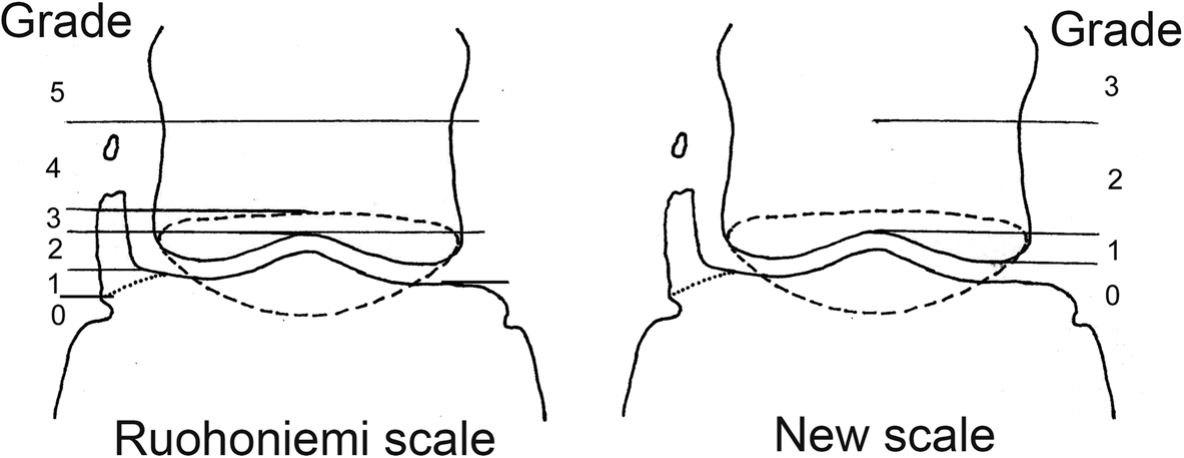
**OUC grading systems.** Grading of ossification according to the Ruohoniemi *et al.* [5] scale and a new scale developed in this study. Left side shows moderate ossification with separate center of ossification and the dotted line is the navicular bone. A separate center of ossification is generally located at a level that results in a grading of 4 or 5. Grading according to Ruohoniemi *et al.* [5]. Grade 0: No ossification, inclination sagittal. Grade 1: Ossification maximum to the distal - palmar level of interphalangeal joint space. Grade 2: To the level of proximal edge of the palmar distal interphalangeal joint space. Grade 3: To a level of the proximal edge of the navicular bone (dotted line). Grade 4: Extending above the navicular bone up to distal half of the middle phalanx. Grade 5: Ossification above distal half of the middle phalanx. New grading according to SLU (new): Grade 0: Ossification not extending proximal of the distal edge of the middle phalanx. Grade 1: Ossification extending between the distal edge of the middle phalanx to a level of proximal edge of the palmar distal interphalangeal joint space. Grade 2 Up to distal half of the middle phalanx. Grade 3: Ossification above distal half of the middle phalanx.

Radiographs were taken on non-sedated horses by different veterinarians at several equine clinics. A dorso-palmar view and horizontal beam was used, with the horse standing on the floor or with the front hooves on 4–8 cm thick wooden blocks. Depending on the facility where the radiographs were taken, the film focus distance varied between 90 and 125 cm and the exposure between 66–75 kV and 3.2–5.0 mAs using a high performance generator and x-ray tube or a portable x-ray unit. For 95% of the horses, conventional film and a cassette with intensifying screens were used, while digital radiography was used for the remaining 5%. All radiographs were blind-coded and evaluated twice by the same person (OW) using both the scale of Ruohoniemi *et al.* [5] and a new scale (NS) in which the navicular bone and the palmar level of the distal interphalangeal joint were excluded as references (Figure 1). The cartilage with the highest grade of ossification, including both the left and right front feet, determined the total score for an individual horse.

Separate centers of ossification (kernels) were registered. However, we had to exclude registration of kernels on two radiographs since radiological examination technique and artifacts, such as small amounts of mud on hooves, made it impossible to be sure if small separate centers of ossification was present or not. Incomplete fusion lines were present in a few horses but not possible to register nor evaluate by radiography in a consistent way and therefore not included in this study.

All official sports data originate from strict protocols kept by groups of officials in trotting associations who use cameras, stopwatches and measuring devices for their evaluations. Swedish data from before 1995 are not digitized and hence were retrieved from microfilmed protocols. Unofficial races and non-competitive (qualification) races were excluded because they were considered to be a source of unreliable data or submaximal performance.

The following data was extracted: number of starts, time record (i.e. best average time over 1 km regardless of distance), career earnings and earnings/race in Swedish/Norwegian Crowns and races completed without official records of slightly irregular trot or breaking into a gallop or pace. The gait data were used to evaluate possible subclinical manifestations of OUC only detectable at high-speed work. Earnings in two different currencies (Swedish and Norwegian Crowns) were comparable [18] over the 35-year-period, about three generations [19,20], of breeding and racing covered in this study.

Body size score (height + chest circumference at withers) of 100 horses, over 3-years-old, from both Sweden and Norway, was either measured by us using stick and tape or taken from official pre-breeding examination data using same methods [14].

### Statistical methods

Data on diagnosis of OUC were of an ordinal nature and were obtained using the two scales (Figure 1). To account for the use of four measurements on each horse, i.e. the lateral and medial grade of ossification in each front hoof, generalized linear mixed (GLM) models [21-24] were used for the analyses. The response variable was modeled using ordinal logistic models with a multinomial distribution and a cumulative logit link function. The horse was used as a random factor. The SAS [23] procedure Glimmix was used for these analyses. Evaluation of the number of races during the career completed without recorded irregularities in gait was made in the GENMOD procedure with a negative binominal distribution in log-transformed (interval 0–1) form. The variable “total earnings in career” displayed a rather skewed distribution and to reduce the effect of possible outliers, this variable was analysed in log-transformed form. Significance level was set at *P* < 0.05.

## Results

The mean and median BLUP values were both 105 among the 648 horses examined (Table 1). The corresponding values were 104 and 105 among horses with and without OUC, respectively, and 107 and 100 among raced and unraced horses, respectively (Table 1).

**Table 1:** **Data for mares, stallions/geldings and all horses included in the study**

**Number of horses included**	**293 mares**	**355 stallions/geldings**	**All 648 horses**
Best linear unbiased prediction value (BLUP)	104	105	105
Mean no. of races throughout the career	23	48 (*)	36
Record (average time in minutes and s/km)^1^	1.33,2	1.30,6 (*)	
Total earnings/career [SEK/NOK]^1^	58,200	228,500 (*)	
Average earnings/start [SEK/NOK]	2,530	4,760 (*)	4160
% of races fulfilled without official record of irregular gait, galopp or pace.^1^	54	59	
% of races with official record of irregular gait^1^	1.5	1.5	
Horses with separate centers of ossification	20	23	43
Average body size score (cm) for the 100 horses measured	348	349	

^1^Data for the 186 mares and 267 stallions/gelding that had competed in official races. *Significant difference (*P* < 0.05).

267 stallions or geldings and 186 mares had raced and these 453 horses completed a total of 23,583 official races, giving a mean of 52 races (median 65) per horse that had raced. There was no significant difference between horses with and without OUC and between horses with different grades of OUC as regards whether they started at all, or number of starts among horses with a racing carrier. Of the 648 horses (2592 cartilages) separate centers of ossification were present in a 43 horses (88 cartilages). Presence of separate centers of ossification did not have significant effect on any of performance parameters.

Regardless of OUC, mares had raced significantly less than stallions and geldings (*P* < 0.0001) and had significantly lower best average time over 1 km (*P* < 0.0001), career earnings (*P* < 0.0001) and earnings/race (Table 1).

The incidence of OUC evaluated with the Ruohoniemi *et al.* [5] scale and the NS is shown in Table 2. Since the scale of Ruohoniemi *et al.* [5] was found to be less specific than the NS in its range 0–2 [13], the incidence of OUC not related to gender (*P* = 0.09) is only presented for the NS (Table 2).

**Table 2:** **Grading of ossification of ungular cartilages in relation to grading system and gender**

**Grade RS 0–5 NS 0–3**	**0**	**1**	**2**	**3**	**4**	**5**
All 648 horses (RS)	169 (26%)	207 (32%)	154 (24%)	49 (7.5%)	39 (6%)	30 (4.5%)
All 648 horses (NS)	454 (70%)	97 (15%)	67 (10%)	30 (5%)	_	_
Males 355 horses (NS)	263 (74%)	45 (13%)	32 (9%)	15 (4%)	_	_
Mares 293 horses (NS)	191 (65%)	52 (18%)	35 (12%)	15 (5%)	_	_

Grade of OUC according to the Ruohoniemi *et al.* [5] scale (RS) and the new scale (NS).

Average number of starts was not affected by any grade of OUC (*P* = 0.98). Furthermore, it did not matter whether OUC was present in the lateral, medial or both sides of a hoof or in one or both hooves. In addition, average race running time was not related to any grade of OUC (*P* = 0.21) or to the medial (*P* = 0.56) or lateral (*P* = 0.64) presence of OUC.

Total earnings in career were significantly higher if the horse had lateral OUC on both the left (*P* < 0.0001) and right front hoof (p = 0.018), but not when the OUC was medial (*P* = 0.94 and *P* = 0.62, for left and right hoof respectively). There was also a significant positive correlation between amount of money won by horses and median lateral OUC (*P* = 0.004), but not median medial OUC (*P* = 0.38). However, when the amount of money won was log-transformed, the overall effect of OUC was no longer significant (*P* = 0.77) and the relationship with median medial (*P* = 0.63) and lateral (*P* = 0.60) OUC formation was non-significant. This suggests that the significant results seen in the untransformed data were caused by a small number of lateral OUC outliers with very large winnings.

Percentage of races without official pace deviations was not significantly related to gradee of OUC (*P* = 0.28). However, horses with OUC grade 1 and 2 according to NS had a tendency (*P* = 0.08) for more frequent records of irregular trot than horses without OUC or OUC of grade 3.

## Discussion

The average BLUP index of 105 indicates that the horses studied accurately represented the Scandinavian population during the study period. As expected, the geldings and stallions were significantly stronger in general performance, but no gender differences were found in OUC or body size. The highly significant gender differences concerning performance data are already often compensated for, by separate races or 20 meters reduced racing distance for mares, when racing against males in Sweden as well as in Norway.

The use of objective official sports data for retrospective analysis is well established [25] but unfortunately objective data were not available for other breeds with a high incidence of OUC, which made comparison between breeds impossible. Between 1973 and 2009 the cold-blooded trotter has through selected breeding become both faster and more rhythmical when trotting at high speed. Andersson *et al.* [26] have identified mutations in certain gene affecting locomotion in many breeds including the cold-blooded trotter. This new research in equine genetics open new possibilities to distinguish physiological from pathological conditions causing gait irregularities in high speed trotting horses. Official reporting of irregular trotting is a subjective method used for improving animal welfare. Since 2009 irregular trotting is no longer reported officially in Sweden but it is still reported in Norway.

Unless fractured, OUC has been questioned as an obvious cause of lameness in several studies [27,28], but it has been suggested by Ruohoniemi *et al.* [8] that it may have an impact on gait at high speed. When grouped together, horses with OUC of grade 1 and 2, according to the NS, had a tendency for more frequent records of irregular trot than horses without OUC or OUC of grade 3 (*P* = 0.08). The gait in cold-blooded trotters was at the time of this study often irregular by nature but, as mentioned above, it has improved over the years through selected breeding, Therefore the tendency for getting an official record when having OUC of grade 1 or 2 should not be exaggerated, irrespective of what may have caused a slightly irregular gait.

Our results do not support theories regarding possible significant ossification [8-11] since official records showed no significant correlation to presence of OUC. In fact, OUC did not in any general way decrease the race performance in the population examined, nor did it cause reported poor performance by gait irregularities at high speed trotting. Hence, our hypothesis that OUC somehow affects the performance of cold-blooded trotters had to be rejected.

Anecdotal evidence sometimes suggests that OUC can cause lameness if the horse is lunged or worked on a volte, especially when highly asymmetrical OUC is present [11]. However, training and racing of cold-blooded trotters are often performed on banked roads or left-handed tracks with cambered curves designed for the higher speed of standard-bred racehorses. The forces created by this way of uneven loading probably far exceed those present when lunging and do not seem to have different effects on horses with or without OUC regardless of body size, separate centers of ossification and regardless which ungular cartilage, lateral or medial, was ossified. Dyson *et al.* [29] reported a positive correlation between extensively ossified cartilage and distal interphalangeal collateral ligament desmopathy diagnosed by clinical findings and MRI. However, it is not clear from their study how they limited the effect of the magic angle [30] on the collateral ligament. Furthermore, adaptation to workload and strain on hooves at extreme speed and for long duration are not taken into consideration in any study on riding sport horses or draught horses. Actually, pathological and physiological conditions can develop separately or simultaneously in the young, growing, athletic horse and this fact must be taken into consideration when evaluating clinical findings in the adult horse.

It has been suggested that both the size and sex of the horse and the limb conformation influence whether and when in life OUC occurs [1,31]. OUC is a moderate to highly inherited condition manifesting itself early in life [4] and is probably triggered by still unknown individual or environmental factors. Today the cold-blooded trotter is trained and raced earlier in life and further studies on ≤2,5 year old individuals, using both pathology and established non-invasive diagnostic equipment such as MRI may provide additional and more relevant information on whether environmental factors affect the development of OUC or not. Furthermore, modern objective gait analysis systems may provide useful information about possible effects of OUC in high speed trotting on straight stretches as well as in banked curves.

From a breeding perspective, both lameness and OUC will occur, but our results strongly question whether OUC alone causes clinical or subclinical lameness in any way that affects performance or animal welfare. Thus compulsory radiological pre-breeding examination of stallions or mares possible grade of OUC should be of a low priority.

## Conclusions

Results from this study can assist many equine professionals in evaluating and interpreting the clinical relevance of radiological findings on ossified hoof cartilage among heavy and high-performing horses. Ossification of ungular cartilages in front hooves of cold-blooded trotters is, regardless of grade or position, not likely to cause decreased performance.

## Abbreviations

BLUP: Best linear unbiased prediction value
kV: Kilovolt
m/s: Meters per second, trotting pace
mAs: Milliampere second
MRI: Magnetic resonance imaging
NS: New suggested radiological scale of OUC evaluation
OUC: Ossification of ungular cartilages
SE: Standard deviation
